# Fibroblast growth factor 9 activates fibroblast activation and drives the progress of shoulder stiffness

**DOI:** 10.3389/fcell.2025.1731453

**Published:** 2025-12-30

**Authors:** Jian Xu, Weihan Yu, Yunkang Kang, Dongqiang Yang, Yanlong Liu, Wenzhi Bi, Haiyang Yu, Beijie Qi, Biao Guo

**Affiliations:** 1 Department of Orthopaedics, Sports Medicine and Arthroscopy, Fuyang People’s Hospital Affiliated to Anhui Medical University, Fuyang, Anhui, China; 2 Department of Sports Medicine, Shanghai General Hospital, Shanghai Jiao Tong University School of Medicine, Shanghai, China; 3 Department of Orthopedics, Shanghai Pudong Hospital, Fudan University Pudong Medical Center, Shanghai, China

**Keywords:** FGF9, fibrosis, PI3K/AKT, shoulder stiffness, transcriptomics

## Abstract

**Background:**

Shoulder stiffness (SS) is a common disease that causes pain and restricted range of motion (ROM), involving synovial inflammation and joint capsule fibrosis. The specific pathogenesis of SS remains unclear. This study aimed to delineate the key molecular driving capsule fibrosis in SS.

**Methods:**

Joint capsule samples from SS and non-SS patients were collected, and high-throughput RNA sequencing along with bioinformatic analysis were performed. A mouse SS model was established via joint immobilization. Functional and immunofluorescence assay were conducted on NIH3T3s. LY294002 was used both in NIH3T3s and mouse SS models.

**Results:**

Transcriptomic analysis identified 100 differentially expressed genes (DEGs). Among the top hub genes, FGF9 was notably upregulated in the SS capsules. *In vitro*, FGF9 promoted NIH3T3s migration, proliferation, and α-SMA expression, effects that were reversed by LY294002. *In vivo*, intra-articular LY294002 injection reduced capsule thickening, fibrosis, and improved passive ROM in SS mice.

**Conclusion:**

Our findings revealed that FGF9 drove fibroblast activation and joint capsule fibrosis in SS via the PI3K/Akt signaling pathway. Targeted inhibition of the PI3K/Akt signaling might represent a promising therapeutic strategy for SS.

## Introduction

Shoulder stiffness (SS) is a prevalent sports medicine disease that causes pain and reduction in range of motion (ROM) (Cole et al., 2009; Kim et al., 2021). The prevalence of SS in the general population is 6.3% (Lahti et al., 2025). SS has a high incidence in middle-aged and elderly women, and exhibits an even higher incidence in diabetic populations, approximately 30% (Cole et al., 2009; Cogan et al., 2022).

The pathogenesis of SS remains unclear (Kim et al., 2025). Risk factors include gender, age, obesity, smoking, diabetes and thyroid disorders (Lahti et al., 2025; Cogan et al., 2022). Given that SS greatly affects patients’ quality of life and increases social burden, investigating the pathogenesis of SS has significant clinical value (Cole et al., 2009; Yan et al., 2023; Bunker et al., 2000).

SS progression includes three classic phases: pain phase, stiffness phase and recovery phase. The stiffness phase often lasts as long as the recovery phase (Reeves, 1975). Inflammatory responses and pathological fibrosis are recognized as the principal pathological processes driving the onset and progression of SS.

Fibroblasts play a pivotal role in modulating cellular microenvironments (Akbar et al., 2019; Liu et al., 2025; Cheng et al., 2025). However, aberrant production of immunomodulatory molecules by fibroblasts may paradoxically drive chronic persistent inflammation (Buckley et al., 2001; Liu et al., 2020; Sun et al., 2023). In diverse pathological conditions, fibroblasts undergo pathological activation, driving the development of fibrotic disorders (Zhang et al., 2023a; Xiang et al., 2025). Multiple studies have showed that persistently activated fibroblasts excessively deposit collagen, leading to fibrosis and progressive dysfunction in various organs (Biggs et al., 2025; Dai et al., 2025; Deng et al., 2025; Li et al., 2025).

Fibroblasts regulate both inflammatory and fibrotic responses in SS pathogenesis (Akbar et al., 2019). The expressions of inflammatory mediators and fibrosis-related cytokines were changed, and the chemotaxis, proliferation and functions of fibroblasts were regulated (Bunker et al., 2000). Fibroblasts may undergo activation through stimulation by factors such as TGF-β, IL-11, and TNF-α (Li et al., 2025; Han et al., 2025; Widjaja et al., 2022; Brokopp et al., 2011). However, the mechanisms underlying fibroblast activation in SS remain unclear.

In this study, we collected the joint capsules of patients with and without SS. Transcriptomic analysis identified fibroblast growth factor 9 (FGF9) as a vital factor inducing fibroblast activation in SS. Targeting the downstream signaling of FGF9 could be a potential therapeutic approach for SS.

## Materials and methods

This study was approved by the Ethics Committee of the Fuyang People’s Hospital (2,024,155). Written informed consent was collected pre-operatively. Animal experiments were approved by the Institutional Animal Care and Use Committee of Shanghai Pudong Hospital (2025-D-G-083).

### Patient inclusion, MRI evaluation and capsule collection

Patients were allocated into SS and non-SS (NC) groups according to published criteria (Chen et al., 2010a). The loss of ROM greater than 25% was defined as SS (Sun et al., 2018). Patients with rotator cuff tears were allocated into NC group.

MRI evaluation was performed using a 1.5 T Magnetic resonance scanner (UNITED IMAGING, uMR560) with an interval of 3 mm for each sagittal slice.

Shoulder capsule samples were obtained following standardized procedure: (Cole et al., 2009): Patient was placed with the affected shoulder abducted at 60° (Kim et al., 2021). Standard arthroscopic portals were established, followed by glenohumeral joint exploration and appropriate debridement (Lahti et al., 2025). Tissue was harvested from the joint capsule using the basket forceps. The sample was immediately transferred into liquid nitrogen.

### Establishment of mouse SS model

8-week-old male C57BL/6J mice were randomly allocated to NC group, Model group, and LY294002 group (4 mouses for each group). The SS model was constructed by joint immobilization for 3 weeks (Qi et al., 2025). Mice in Model group and LY294002 group received intra-articular injections of 20 μL PBS or LY294002 (1.5 mg/mL) at one and 2 weeks postoperatively. NC group received sham surgery. Mice were kept under a 12 h light/dark cycle and were free to food and water. All animals were euthanized by overdose CO_2_ 3 weeks postoperatively. Shoulder joint samples were then collected. ROM assessment was performed using a published methodology (Luo et al., 2022).

### High-throughput RNA sequencing

Total RNA was extracted from 12 capsules (6 SS and 6 NSS). Agilent 4,200 Bioanalyzer was used to assess the quality and size distribution of cDNA libraries. Sequencing was performed and data were analyzed using Seqtk (v1.3) and aligned to the GRCh38/hg38 reference genome. Transcript quantification was carried out and normalized. Data was analyzed by EdgeR (v3.42.0) in R (v4.4.3). Differentially expressed genes (DEGs) were defined at |log_2_FC| > 1 and p < 0.05.

### Acquisition and analysis of public transcriptomic data

Gene expression data related to adhesive capsulitis (AC) were obtained from the Gene Expression Omnibus (GSE140731), comprising 48 samples (26 AC cases and 22 controls). Data analysis was conducted using the same statistical criteria (|log_2_FC| > 1 and p < 0.05) to identify significant DEGs.

### Functional and pathway enrichment analysis

Gene Ontology (GO) enrichment and Kyoto Encyclopedia of Genes and Genomes (KEGG) pathway analyses were performed (Mu et al., 2024). The lists of DEGs from SS vs. NSS, AC vs. control, and the common DEGs between them were submitted separately. For the common DEGs, GO enrichment was conducted separately for the upregulated and downregulated gene subsets.

### Protein-protein interaction (PPI) network construction and module analysis

The common DEGs was submitted to the STRING database. A minimum interaction score of 0.4 was set. The resulting network was imported uploaded to Cytoscape (v3.9.1). The Molecular Complex Detection (MCODE) plugin was used to identify highly interconnected clusters.

### Hub gene screening and diagnostic value evaluation

The top 5 candidate hub genes were determined by aggregating the results from all 12 calculation algorithms available in cytoHubba. The Receiver Operating Characteristic (ROC) curve was constructed and the Area Under the Curve (AUC) was calculated.

### Cell culture and intervention

Murine fibroblast NIH3T3s were purchased from Servicebio. Cells were maintained in high-glucose DMEM medium with 10% fetal bovine serum +1% penicillin/streptomycin at 37 °C with 5% CO_2_. For *in vitro* experiments, cells were treated with FGF9 or LY294002 of 50 ng/mL and 3 μg/mL for 24 h, respectively.

### Wound healing assay

When cells reached 80% confluence, a linear scratch was created by a pipette tip. Then, different interventions were administered for 24 h. After that, cells were observed and imaged under a microscope. The migration ability was quantified according to the recovery rate.

### EdU assay

Cells were labeled with EdU solution for 2 h. Next, cells were fixed and permeabilized. After that, click reaction regent was employed. Finally, images were acquired using fluorescence microscope.

### Hematoxylin & eosin (HE) and masson staining

Joint samples were embedded and sectioned at 8 μm. Then, sections were stained with hematoxylin and eosin for 5 min each. Masson staining was conducted using a commercial kit (G1006, Servicebio). Images were captured by microscope.

### Immunofluorescence staining

Sections were treated with antigen retrieval solution (G0142, Servicebio) and blocking buffer (P0102, Beyotime). NIH3T3s were fixed and blocked. Next, sections and cells were incubated with corresponding primary and secondary antibodies. DAPI was used to locate nuclei. Images were captured by fluorescence microscope.

### Statistical analysis

GraphPad Prism was used for data analysis. Data was presented as mean ± SD. Student’s t-test and one-way ANOVA were employed for comparisons. P < 0.05 was considered as statistical significance.

## Results

### SS patients exhibited significant capsule fibrosis and impaired shoulder function

**FIGURE 1:**
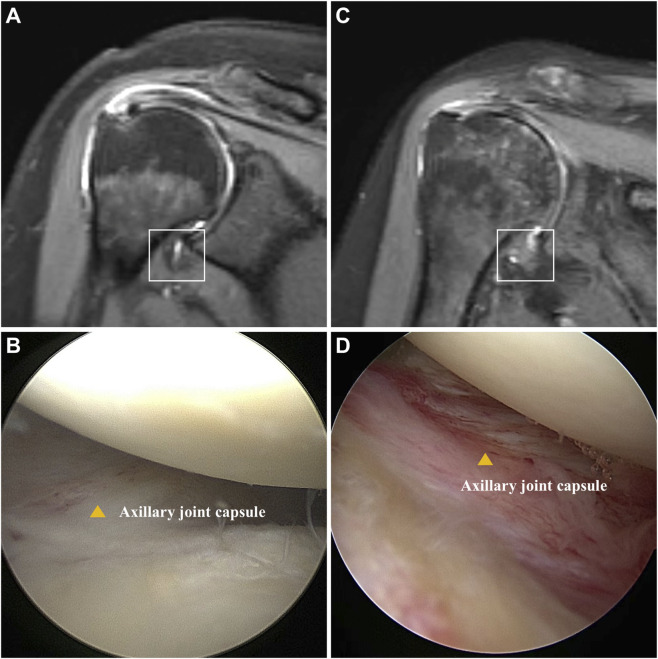
Comparison of patients with and without SS. **(A)** Oblique coronal fat-suppressed T2-weighted MRI shows no thickening or edema of the humeral joint capsule in the axillary recess (square). **(B)** Arthroscopy revealed no hyperplasia or redness and swelling in the axillary joint capsule (yellow triangle). **(C)** Oblique coronal fat-suppressed T2-weighted MR image showed thickening and edema of joint of axillary recess (square). **(D)** Arthroscopy revealed hyperplasia and redness of the axillary capsule of the shoulder joint (yellow triangle).

A total of 12 patients (6 for SS and 6 for non-SS) were included in this study, and the shoulder joint capsules were collected. The baseline data and ROM measurement results were demonstrated in Table 1. There was no significant difference in gender, age, BMI, or symptom duration between SS and non-SS groups. However, a significantly decrease of ROM was observed in SS patients, indicating joint stiffness. Additionally, there were higher VAS scores and lower Constant scores in the SS group, reflecting severe pain and functional impairment. MRI results showed significant thickening and edema in the capsule of SS patients (Figures 1A,C). Pronounced capsule hyperplasia and swelling were observed in the SS group under arthroscopy (Figures 1B,D). These results suggested that there were significant joint stiffness, capsule fibrosis and function impairment in SS patients.

**TABLE 1:** Basic characteristics and shoulder measurements of analyzed patients.

Items	Without SS	SS	P value
Gender (M:F)	1:5	1:5	1.000
Age	59.6 ± 10.4	56.3 ± 9.8	0.581
Body mass index	23.9 ± 2.9	22.2 ± 2.4	0.295
Duration (month)	3.9 ± 1.9	4.7 ± 1.5	0.42
Flexion	175.5 ± 3.7	104.3 ± 20.5	<0.001^a^
Abduction	175.3 ± 3.2	95.6 ± 22.9	<0.001^a^
External rotation	71.6 ± 6.2	13.3 ± 9.8	<0.001^a^
VAS	3.8 ± 0.9	4.8 ± 0.8	0.075
Constant score	52.1 ± 4.3	43.9 ± 5.2	<0.05^a^

^a^ Statistical significance.

### Transcriptomic analysis identified FGF9 as a potential pathogenic molecule in SS

**FIGURE 2:**
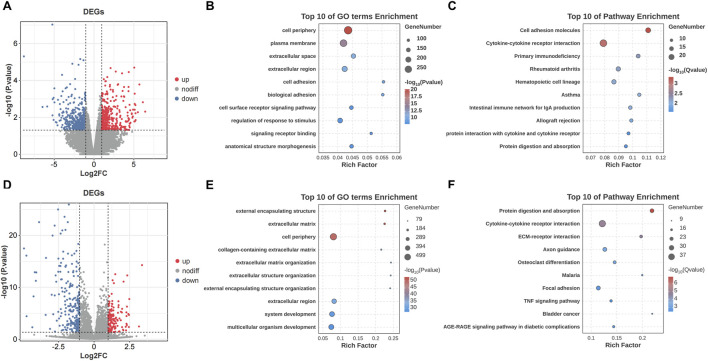
Transcriptomic profiling and functional enrichment of DEGs in SS and AC. **(A)** Volcano plot of DEGs between the SS and NSS groups. **(B)** GO enrichment analysis of DEGs in SS. **(C)** KEGG pathway enrichment analysis of DEGs in SS. **(D)** Volcano plot of DEGs between the AC and control groups. **(E)** GO enrichment analysis of DEGs in AC. **(F)** KEGG pathway enrichment analysis of DEGs in AC.

The transcriptomic analysis on the patients’ capsules identified 821 DEGs, with 375 upregulated and 446 downregulated. The result was demonstrated by volcano plot (Figure 2A). GO analysis showed that DEGs were enriched in “cell periphery”, “plasma membrane” and “extracellular space” (Figure 2B). KEGG pathway analysis indicated that DEGs were enriched in “Cell adhesion molecules” and “Cytokine-cytokine receptor interaction” (Figure 2C). In addition, the transcriptomic analysis between AC and negative controls identified 1,025 DEGs, with 692 upregulated and 333 downregulated (Figure 2D). The results of GO and KEGG analysis were shown in Figures 2E,F.

**FIGURE 3:**
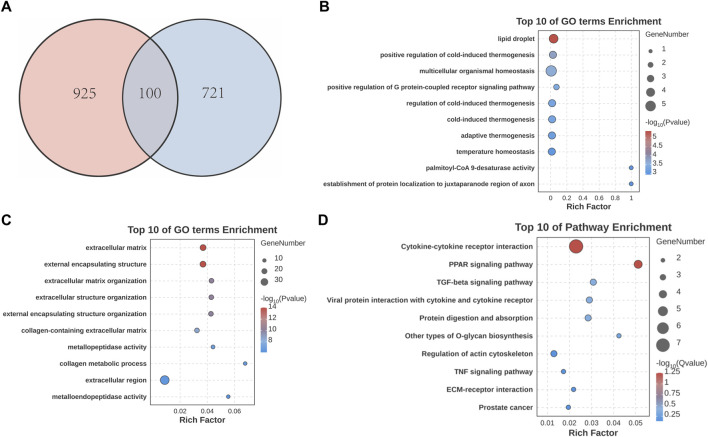
Identification and analysis of common DEGs in SS and AC. **(A)** Venn diagram showing the overlap of DEGs between SS and AC. **(B)** GO enrichment analysis of upregulated common DEGs. **(C)** GO enrichment analysis of downregulated common DEGs. **(D)** KEGG pathway enrichment analysis of the common DEGs.

Next, we conducted cross-analysis based on the datasets between SS and AC, and identified 100 common DEGs with 80 upregulated and 20 downregulated (Figure 3A). GO enrichment showed that DEGs were highly enriched in “extracellular matrix” and “collagen metabolic process” (Figures 3B,C). KEGG analysis showed predominant enrichment in “Cytokine-cytokine receptor interaction” and “TGF-beta signaling pathway” (Figure 3D).

**FIGURE 4:**
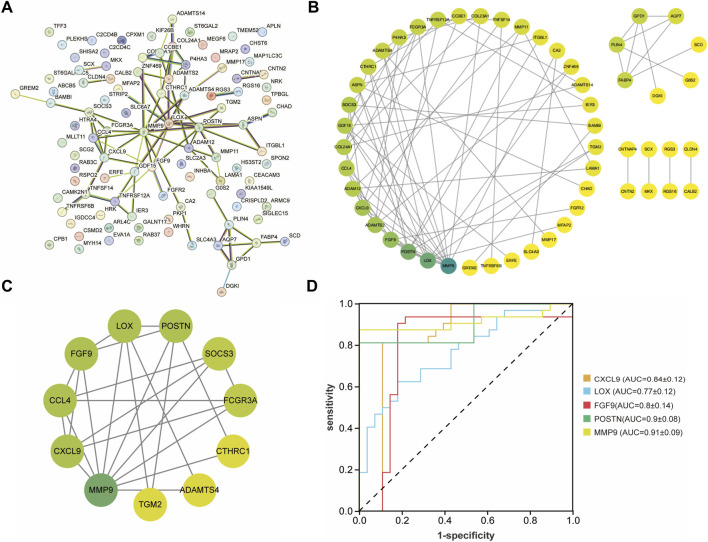
Construction and analysis of the PPI network. **(A,B)** PPI network of the common DEGs. The darker the green color, the higher the degree of nodes. **(C)** Key clustering module of the PPI (the cluster score is 4.8). **(D)** ROC curve of CXCL9, LOX, FGF9, POSTN, MMP9.

After that, these DEGs were imported into the String database to construct a PPI network (Figures 4A,B). The MCODE algorithm was employed to identify key cluster modules and revealed a densely interconnected module consisting of 11 nodes and 24 edges, with a high cluster score of 4.8. These results indicated the robust and biologically significant interactions among these proteins (Figure 4C). To further investigate the interaction relationships among hub genes, the cytoHubba algorithm was applied and identified the top five genes including CXCL9, LOX, FGF9, POSTN, MMP9. Additionally, the ROC curves were generated and the AUC with a 95% CI was calculated (Figure 4D). Among these five genes, FGF9 was selected as the candidate pathogenic molecule.

### FGF9 expression was upregulated in SS capsules

**FIGURE 5:**
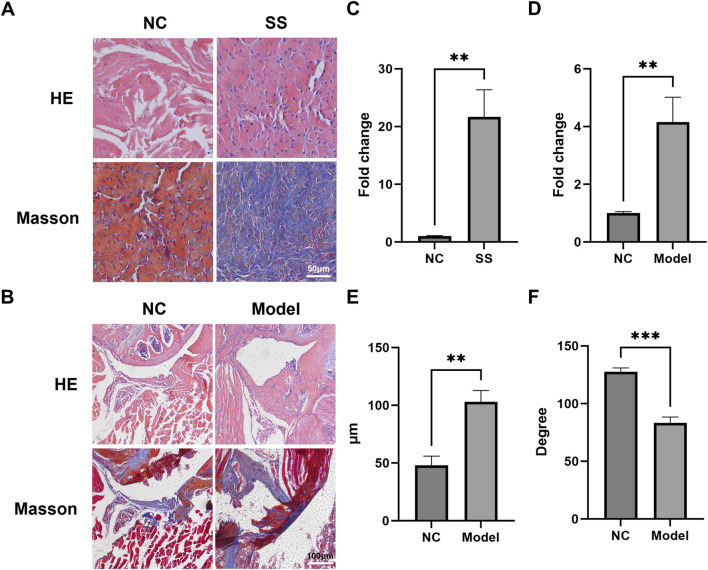
The joint capsules of SS patients and SS mice exhibited significant fibrosis. **(A,B)** Representative HE and Masson images of the capsules of SS patients and SS mice. **(C)** Quantification of collagen deposition in the patient capsules. **(D)** Quantification of cell infiltration in the mouse capsules. **(E)** Average thickness of the mouse capsules. **(F)** Passive ROM of mouse shoulder joint. **p < 0.01, ***p < 0.001.

To validate the multi-omics findings, we performed histological analysis on capsules from SS patients and established a mouse SS model. HE and Masson staining showed that the cell infiltration and collagen expression in SS group was significantly greater than NC group (Figures 5A,C). Similarly, the capsules of mouse SS models also exhibited significantly enhanced cell infiltration and capsule thickening (Figures 5B,D,E). The ROM of SS mice was markedly decreased compared to NC (Figure 5F). These results indicated that both clinical and mouse SS capsules develop characteristic fibrosis.

**FIGURE 6:**
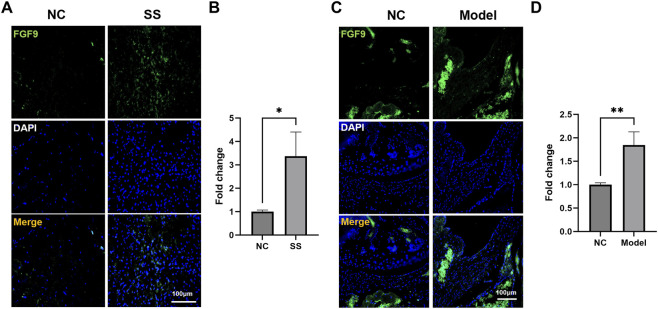
FGF9 expression was upregulated in the SS capsules. **(A,B)** Immunofluorescence images showed FGF9 expression in the patient capsules. **(C,D)** Immunofluorescence images showed FGF9 expression in the mouse capsules. *p < 0.05, **p < 0.01.

Subsequently, we assessed the FGF9 expression in the SS capsules. Immunofluorescence staining showed that the FGF9 were significantly upregulated in the capsules of both SS patients and SS mice (Figures 6A–D). These results aligned with our multi-omics analysis, suggesting that FGF9 played a critical role in SS pathogenesis.

### FGF9 induced fibroblasts activation and capsule fibrosis via PI3K/Akt signaling pathway

**FIGURE 7:**
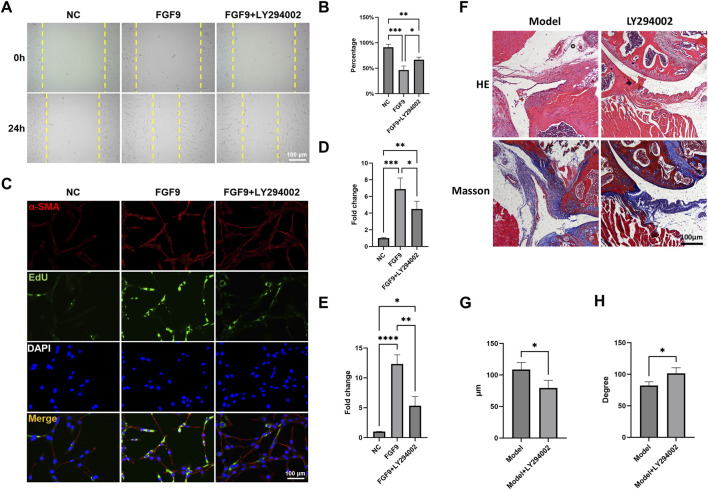
FGF9 induced fibroblasts activation and capsule fibrosis via PI3K/Akt signaling pathway. **(A,B)** Wound healing assay of NIH3T3s with different treatments. **(C)** Immunofluorescence staining and EdU assay demonstrated α-SMA expression and cell proliferation ability in NIH3T3s. **(D)** Quantification of α-SMA expression. **(E)** Quantification of cell proliferation ability. **(F)** HE and Masson staining of the mouse capsules. **(E)** Average thickness of the mouse capsules. **(F)** Passive ROM of mouse shoulder joint. *p < 0.05, **p < 0.01, ***p < 0.001, ****p < 0.0001.

In order to unveil the effect of FGF9 on fibroblasts, we employed LY294002, a specific inhibitor of the PI3K/Akt pathway. Wound healing assay showed that FGF9 significantly enhanced the migration ability of NIH3T3s (Figures 7A,B). Additionally, immunofluorescence staining and EdU assay demonstrated that the α-SMA expression and proliferation activity of NIH3T3s were markedly upregulated (Figures 7C–E). However, this activation was significantly reversed by LY294002.

We further evaluated the therapeutic effect of LY294002 *in vivo*. HE and Masson staining demonstrated that LY294002 significantly reduced capsule thickness when compared to Model group (Figures 7F,G). There was also a significant restore in ROM after LY294002 treatment (Figure 7H). These results indicated that FGF9 induced fibroblasts activation and capsule fibrosis through PI3K/Akt signaling pathway. Targeted inhibition of PI3K/Akt signaling could be a promising therapeutic strategy for SS.

## Discussion

In this study, clinical capsules were collected and mouse SS models were established. The transcriptomic analysis identified FGF9 as a potential pathogenic factor. Experiments validated that FGF9 was significantly upregulated in the SS capsules, and FGF9 induced fibroblasts activation and capsule fibrosis through PI3K/Akt signaling pathway. LY294002 effectively attenuated fibroblasts activity and capsule fibrosis. This study elucidated a novel FGF9/PI3K/Akt axis in SS pathogenesis.

SS is a prevalent shoulder disorder. It has three classic clinical phases: pain stage, frozen stage, and thawing stage (Millar et al., 2022). The main pathological mechanisms of SS are inflammation and fibrosis (Dias et al., 2005; Sun et al., 2021). Starting with inflammation, immune cells accumulate in the capsules and secrete cytokines such as IL-1β, IL-6, and TGF-β (Akbar et al., 2019; Rodeo et al., 1997). Then fibroblasts are recruited and differentiate into myofibroblasts. The activated myofibroblasts excessively synthesize and deposit collagen into ECM, resulting in capsule fibrosis (Qi et al., 2025; Luo et al., 2022). Therefore, it is critical to identify the key factor regulating fibroblast for developing therapeutic interventions for SS.

FGF9 belongs to fibroblast growth factor family. It is a crucial intercellular signaling molecule that widely distributed in the body (Kim et al., 2006; Chen et al., 2010b). By binding to its receptor, FGF9 activates downstream signaling including MAPK/ERK and PI3K/Akt (Chen et al., 2024; Tang et al., 2021), and exerts various biological functions related to skeletal development, angiogenesis, apoptosis and neural regeneration. For example, Zhang et al. demonstrated that FGF9 significantly promoted hepatocellular carcinoma by recruiting β-catenin to increase hepatic ECM accumulation (Zhang et al., 2023b). Meanwhile, FGF9 plays an important role in fibrogenesis, as evidences showed that FGF9 was upregulated in various fibrotic diseases (Zhang et al., 2023a). FGF9 can directly activate fibroblasts and lead to tissue fibrosis (Joannes et al., 2016). In this study, multi-omics analysis identified FGF9 as a potential pathogenic molecule in SS. We subsequently validated that FGF9 was significantly upregulated in capsules of both SS patients and SS mice. *In vitro* experiments showed that FGF9 markedly enhanced the activity of fibroblasts and elevated α-SMA expression. These findings suggested that FGF9 played an important role in SS pathogenesis.

The PI3K/Akt signaling is pivotal in various cellular processes (Guo et al., 2024; Cheng et al., 2024). After being stimulated by upstream molecules, PI3K becomes activated, recruits and phosphorylates its downstream effector—Akt. The Akt then modulates biological processes including proliferation, metabolic homeostasis and immune regulation (Tewari et al., 2022; Nepstad et al., 2020; Ji et al., 2025). The PI3K/Akt is also involved in fibrogenesis by promoting fibroblasts activation and interacting with profibrotic mediators such as TGF-β (Qi et al., 2025; Wang et al., 2022; Tang et al., 2023; Luo, 2017). Huang et al. demonstrated that the PI3K/Akt/mTOR pathway played a critical role in the progression of pulmonary fibrosis by influencing fibroblast activation (Huang et al., 2024). In the SS progression, cytokines such as IL-1β and TNF-α upregulate the FGF9 levels in SS capsules. FGF9 then activates its receptor FGFR, which in turn stimulates the PI3K/Akt signaling pathway and activates fibroblast, leading to excessive ECM deposition and driving fibrosis. In this study, we found that FGF9 significantly increased fibroblasts activity, while LY294002 remarkably attenuated this FGF9-induced activation. Moreover, LY294002 effectively mitigated capsule fibrosis and restored ROM *in vivo*. Together, these results indicated that FGF9 had the regulatory effect on fibroblasts activation and capsule fibrosis through PI3K/Akt signaling pathway. Targeting PI3K/Akt might be a promising therapeutic approach for SS.

This study has several limitations. First, the mouse SS model was established by joint immobilization in healthy mice. Given the clinical association between SS and metabolic disorders (Ramirez, 2019), future research employing disease-specific models, such as diabetic mice. Second, while we identified PI3K/Akt as a critical pathway, other parallel pathways might also be activated by FGF9 and contributed to fibrotic process. This needs further investigation in the future. Third, direct use of LY294002 *in vivo* may cause toxic reactions. Exploring the therapeutic approaches combined with biomaterials such as drug-loaded liposomes for better biocompatibility will enhance the value of clinical translation.

## Conclusion

In this study, we identified FGF9 as a potential pathogenic factor in SS through transcriptomics. Experimental validation demonstrated that FGF9 induced fibroblast activation and capsule fibrosis via the PI3K/Akt signaling pathway. Targeting the PI3K/Akt signaling held the potential to become a promising therapeutic strategy for SS.

## Data Availability

The raw data supporting the conclusions of this article will be made available by the authors, without undue reservation.
